# Performance Evaluation of Modified Rubberized Concrete Exposed to Aggressive Environments

**DOI:** 10.3390/ma14081900

**Published:** 2021-04-11

**Authors:** Akram M. Mhaya, Mohammad Hajmohammadian Baghban, Iman Faridmehr, Ghasan Fahim Huseien, Ahmad Razin Zainal Abidin, Mohammad Ismail

**Affiliations:** 1 School of Civil Engineering, Faculty of Engineering, Universiti Teknologi Malaysia, Skudai 81310, Johor, Malaysia; akrammhaya1985@gmail.com (A.M.M.); mohammad@utm.my (M.I.); 2 Department of Manufacturing and Civil Engineering, Norwegian University of Science and Technology (NTNU), 2815 Gjøvik, Norway; 3 Institute of Architecture and Construction, South Ural State University, Lenin Prospect 76, 454080 Chelyabinsk, Russia; faridmekhri@susu.ru; 4 Institute for Smart Infrastructure and Innovative Construction, School of Civil Engineering, Faculty of Engineering, Universiti Teknologi Malaysia, Skudai 81310, Johor, Malaysia; fhghassan@utm.my

**Keywords:** acid and sulphate attacks, elevated temperatures, GBFS, rubberized concrete, WRTCs

## Abstract

Recycling of the waste rubber tire crumbs (WRTCs) for the concretes production generated renewed interest worldwide. The insertion of such waste as a substitute for the natural aggregates in the concretes is an emergent trend for sustainable development towards building materials. Meanwhile, the enhanced resistance of the concrete structures against aggressive environments is important for durability, cost-saving, and sustainability. In this view, this research evaluated the performance of several modified rubberized concretes by exposing them to aggressive environments i.e., acid, and sulphate attacks, elevated temperatures. These concrete (12 batches) were made by replacing the cement and natural aggregate with an appropriate amount of the granulated blast furnace slag (GBFS) and WRTCs, respectively. The proposed mix designs’ performance was evaluated by several measures, including the residual compressive strength (CS), weight loss, ultrasonic pulse velocity (UPV), microstructures, etc. Besides, by using the available experimental test database, an optimized artificial neural network (ANN) combined with the particle swarm optimization (PSO) was developed to estimate the residual CS of modified rubberized concrete after immersion one year in MgSO_4_ and H_2_SO_4_ solutions. The results indicated that modified rubberized concrete prepared by 5 to 20% WRTCs as a substitute to natural aggregate, provided lower CS and weight lose expose to sulphate and acid attacks compared to control specimen prepared by ordinary Portland cement (OPC). Although the CS were slightly declined at the elevated temperature, these proposed mix designs have a high potential for a wide variety of concrete industrial applications, especially in acid and sulphate risk.

## 1. Introduction

Nowadays, sustainable development and environmental safety are two topics that are of paramount concern to researchers, particularly within developed nations. The materials used in the construction sector will have a direct effect on energy consumption, CO_2_ emissions, conservation of natural resources, and landfill waste produced by a building project [1,2]. Several ecological issues are central to the construction materials industry, including the rapid depletion of the river sand and crushed gravel-based natural aggregate and overexploitation of the ordinary Portland cement (OPC), leading to the release of excess amounts of carbon dioxide. Additionally, the construction materials industry is associated with the problems surrounding waste production and disposal, linked to landfill space scarcity [3,4,5,6].

In the construction industries worldwide, concretes have extensively been utilized over the years [7]. Every unit volume (m^3^) of the concrete contains around 0.6 to 0.7 m^3^ of the fine and coarse aggregates. This is both due to their widespread accessibility, cost-effectiveness, and simplicity of their characteristics. Nevertheless, conventional concrete often fails to demonstrate appropriate durability when encountering aggressive environments, such as those with high levels of sulphates or acidity. OPC based concrete does not show resistance to acidity due to the presence of calcium compounds where they are easily dissolved in an acidic environment, resulting in increased porosity and rapid deterioration [8,9]. Furthermore, conventional concrete production requires a large amount of aggregate, resulting in the rapid depletion of natural aggregate resources. Therefore, discovering new, or potentially synthetic, aggregates that might be used as an alternative to natural aggregates is crucial and, thus, the subject of ongoing research.

Using waste rubber tire crumbs (WRTCs) instead of natural aggregates within civil engineering emerged as one solution to environmental issues. The WRTCs has been utilized in different kinds of concrete where the results indicated that the compressive strength (CS) and elastic moduli of the concretes showed a reduction and strongly depended on the rubber contents [10,11,12,13]. The further parametric analysis demonstrated that rubber particles could only compose up to 20% of the aggregates’ total composition before a large drop in the concrete’s strength occurs [14]. Turatsinze et al. [15] found that a rubber surface formed a weak bond with the cement paste due to its hydrophobic nature. Therefore, several researchers suggested that the bonding among cement pastes and particles of the WRTCs can be improved either by treating the rubber aggregates with the NaOH solution or adding supplementary cementitious materials (SCMs) [16,17,18]. Turki et al. [19] also demonstrated that the mechanical properties of the rubberized concretes could be improved through the combination of mineral fillers, such as siliceous or limestone, with the rubber.

Granulated blast furnace slag (GBFS) is an industrial by-product of steel manufacturing, frequently utilized as supplementary cementitious material (SCMs) in concrete production to partially substitute for OPC [20,21,22]. In-depth researches on the GBFS modified concretes has suggested that it has a lower heat evolution, less permeability, greater strength over time, and high resistance against the chemical attack [23,24,25,26]. Additionally, [24,25] both report that within 28 days, the compressive strength of GBFS concrete increases as the GBFS replacement ratio increases, up until a 40–60% replacement level, beyond this level, the strength of the GBFS concrete begins to decrease. So far, little attention has been paid to the effects of the GBFS inclusion on the durability properties of the rubberized concretes with WRTCs as the replacement agent to the fine and/or coarse aggregates. From this perspective, the current research examines the durability performance of GBFS inclusion in OPC-based concretes with different content of WRTCs against aggressive environments i.e., chemical attacks, elevated temperature, etc. To evaluate rubberized modified concrete’s durability performance, several parameters including residual CS, elevated temperature resistance, and non-destructive variables (microstructures measurements, ultrasonic pulse velocity (UPV)), were investigated on twelve batches of concrete specimens.

## 2. Materials and Methods

### 2.1. Materials Properties

In this study, to prepare the modified concrete specimens, the pure GBFS representative of SCM was used to replace the OPC and increase the contents of the aluminum silicate (AS) and concrete binders’ chemical composition. The chemical composition of OPC and GBFS was determined using X-ray fluorescence (XRF) spectroscopy. The XRD results shown in Table 1 confirm that the main compounds in OPC and GBFS were CaO, SiO_2,_ and Al_2_O_3_. GBFS presented lower CaO content (51.8%) compared to OPC (67.8%); however, it has a high content of SiO_2_ (30.8%) and Al_2_O_3_ (10.9%) compared to 17.6% and 4.5% obtained with OPC, respectively. It is well known that the SiO_2_, Al_2_O_3_, and CaO contents are highly significant for the hydration reaction to form the C-S-H and C-A-S-H gels. In both OPC and GBFS, it was observed that the total content of oxides Fe_2_O_3_, MgO, and K_2_O was less than 6%. The total loss on ignition (LOI) was found to be lower in GBFS chemical composition (0.2) compared to OPC (1.7). According to ASTM C618 [27], the GBFS contains nearly 93% of calcium, silicate, and alumina that fulfill the pozzolanic material requirement. The specific surface area, specific gravity, and median particle sizes of the GBFS were 4950 cm^2^/g, 2.89 g/cm^3,^ and 12.4 µm, respectively. In the current study, the OPC had the specific gravity of 3.15 g/cm^3,^ which obeyed the specified standard as stated by Shetty [28], ranging from 3.10 and 3.25 g/cm^3^.

The modified concrete specimens were prepared following the ASTM C33 [29], where the natural river sand was utilized as fine aggregates. Before using the sand, the water-cement ratio was obtained by maintaining the sand in saturated surface dry (SSD) condition in compliance with ACI-219 [30]. The absorption ability and specific gravity (2.7) of the sand (graded well with the fineness modulus of 2.8) were examined with SSD condition. The sand’s specific gravity was in the allowable range (2.4 to 3.0) and was somewhat below compared to that of the value of the coarse aggregates [31]. The water absorbance of the river sand (2.2%) was in the referred range (0.2 to 3.2%) [31]. The ground granite stones (devoid of any damaging components like the organic compounds, grasses and leaves, dry mud, and silts) were utilized as the coarse aggregates. The specific gravity, water absorption capacity, and particle size percent of the coarse aggregates were examined under the SSD conditions. In this research, the natural aggregate created 71% of the conventional and GBFS modified concrete’s overall weight in which the largest particle size of the river sand (fine) and crushed stone gravel (coarse) was 4.7 and 10 mm, respectively. The fine and coarse aggregates physical characteristics are shown in Table 2.

**Figure 1:**
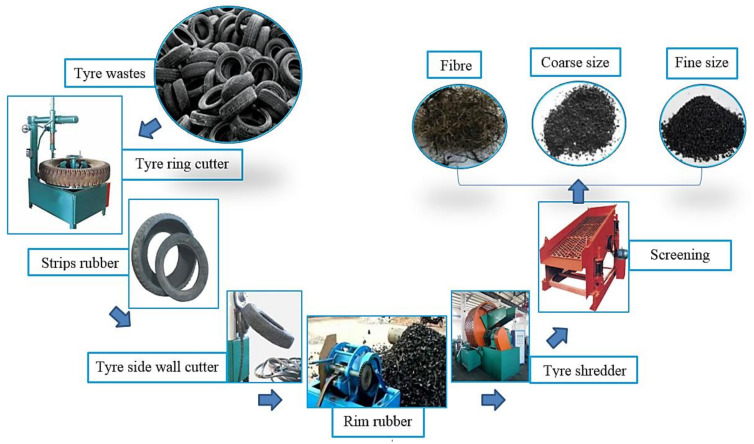
Fine and coarse waste rubber tire crumbs (WRTCs) preparation process.

Figure 1 shows the preparation procedures of the WRTCs where the discarded tires were cut into tiny pieces by the shredders and shearing instrument. The modified rubberized concretes were prepared from 2 types of the WRTCs (graded by ASTM C136), having the grain size ranged from 1–4 mm and 5–8 mm, as shown in Table 2.

Table 3 depicts the physical and chemical properties of the WRTCs used as a substitute for the natural aggregates in the concrete. The hydrocarbon contribution from the rubber was above 50% of the total chemical compositions of the WRTCs.

### 2.2. Mix Design and Specimens Preparation

Using GBFS and WRTCs replacement agents, four batches (A, B, C, and D) were developed as high-performance, sustainable concrete (Table 4). The water to cement ratio (w/c) was fixed to 0.55 for all modified concrete mixes. In batch A, two mixes were prepared, including a cement binder (100% of OPC) and a modified cement binder (OPC was replaced by 20% of GBFS). The concrete mix prepared with 100% OPC and natural aggregates is adopted as control sample to evaluate modified concrete’s performance containing GBFS and WRTCs in aggressive environments. Since the mechanical and durability of the modified concretes were affected by the sizes and contents of the WRTCs aggregates, the B, C, and D batches were developed with different content of WRTCs aggregate. In batch B, the fine WRTCs aggregate was used to replace the river sand by 5, 10, 20, and 30%. Unlike in batch C, river sand content kept constant, and coarse WRTCs aggregate replaced crushed stone with 5%, 10%, 20%, and 30%. In the last batch, the fine and coarse WRTCs incorporating (50:50) and used to replace the river sand and crushed stone with 5%, 10%, 20%, and 30%. Additionally, for all the prepared mixes, the amounts of the binder, water, fine and coarse aggregates were kept constant to 419, 230.5, 721, and 995 kg/m^3^, respectively. The binder of all modified rubberized concrete consists of OPC and GBFS by the ratio of 80% and 20%, respectively. The replacements of the fine, coarse, and fine/coarse aggregates are denoted by RF5-30, RC5-30, and RFC5-30, respectively.

The SSD conditioned river sand, and crushed stone aggregates were blended in the mixer for 2 min to prepare the modified concrete specimens, later the modified cement (50%) and WRTCs aggregate (50%) were added to the blend and again mixed for two more minutes. The remaining 50% of the WRTCs were added gradually with the rest 50% of the modified cement and blended in the mixer for 2 min before hydrated with water. The resultant freshly obtained concretes were cast within the mould in three layers, where the vibration table reinforced each layer for 20 s to remove the air gaps. After the casting was over, the specimens were left 24 h at the ambient temperature of 23 ± 3 °C and relative humidity of 60%. Finally, the obtained concrete specimens were de-moulded and immersed inside water for 7 days following the ASTM C192 [32] procedure before being left under the same ambient laboratory condition till the tests were performed.

### 2.3. Tests Program

The several tests were conducted to evaluate the performance of modified rubberized concrete in the harsh environment compared to the control specimen (Table 5).

The H_2_SO_4_ procured from the QREC (Malaysia) solution of 10% was prepared using the deionised water to evaluate the rubberized concretes’ resistance against the acid attacks. To maintain the constant pH level, the acid solution was closely monitored and changed every 90 days. Quantities such as weight loss, UPV, and residual CS were determined following the ASTM C267 [33] stipulation after ages of 365 days. The sulphate attacks in the rubberized concrete occurred due to the penetration of the (SO_4_)^−2^, Mg, Ca, or Na cations transported into the concrete matrix at different water contents. A similar approach was applied to evaluate the sulphate attack resistance of modified rubberized concrete using10% MgSO_4_ solution.

An ultrasonic pulse velocity (UPV) test is an in situ, non-destructive test to check the quality of concrete in terms of material discontinuities, any damages/cracks, and the level of corrosion for a given exposure time. In this test, the strength and quality of concrete are assessed by measuring the velocity of an ultrasonic pulse passing through a concrete structure (direct method). The pulse velocity can be determined by measuring the length between the transducers and the travel time, Equation (1), in which the faster level of velocity indicates integrity, higher density, and quality of the material.

(1) $$\mathrm{UPV}=v_{c}\left(x,t\right)=x/t$$

where, *x* is distance and *t* is transit time.

The performance of modified rubberized concrete expose to high temperatures was evaluated using an automatic electric furnace. Each cubic specimen was cured for 28-days and then exposed to 500 °C and 900 °C elevated temperature at varying durations, as illustrated in Table 5. Each specimen’s weight was recorded before and after the heat treatment to determine the weight loss due to the elevated temperatures followed by the CS testing. Their residual CS and percentages of the strength loss, surface texture, and UPV were measured according to the ASTM C597 specification [34]. In addition, the microstructures of the modified rubberized concretes were analyzed using the XRD and SEM measurements. The XRD and SEM measurements were also performed to evaluate the rubberized modified concrete’s microstructures, crystallinity, and surface morphologies.

## 3. Results and Discussion

### 3.1. Resistance Against Sulphate and Acid Attacks

**Figure 2:**
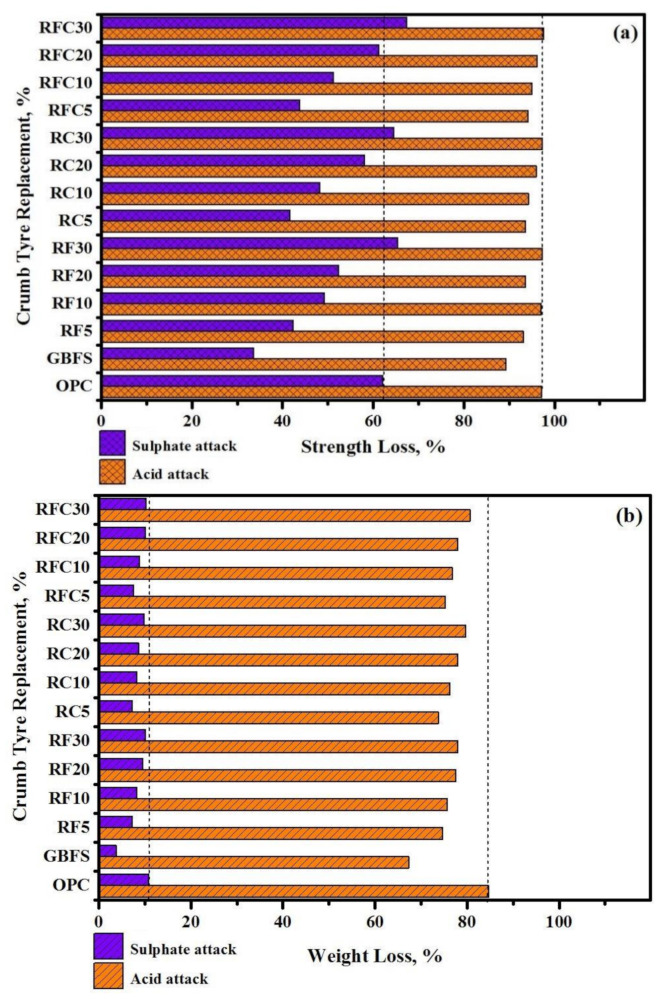
Effect of MgSO_4_ and H_2_SO_4_ on (**a**) compressive strength (CS) and (**b**) weight loss of modified rubberized concrete.

The various tests were conducted to determine the rubberized concrete’s basic degradation mechanism under the exposure of the sulphate and acid solutions. Figure 2 shows the reduction of CS and the weight of all tested specimens after 365-day immersion in the MgSO_4_ (sulphate) and H_2_SO_4_ (acid) solutions. The results indicate that the CS experienced a major reduction by immersion in acid solution compared to sulphate solution (Figure 2a). The average CS reduction in the rubberized modified concrete specimens were immersed in sulphate solution was 53%, while this value was increased to 95% after immersion in acid solution. Such a significant reduction in the CS after immersion in the acid solution can explain by SO_4_^−2^ reaction with the Ca(OH)_2_ that produces CaSO_4_.2H_2_O, leading to an expansion of the paste matrix and extra cracks generation in the concrete.

The results also acknowledged increasing the WRTCs level, either as fine or coarse aggregate, leading to a reduction in the CS in modified rubberized specimens immersed in acid and sulphate solutions. For example, by increasing the WRTCs level from 5 to 30%, the CS of specimens attacked by sulphate and acid solutions were reduced by an average of 55% and 4%, respectively. The lower bond strength between the rubber aggregates and cement pastes resulted in the gypsum formulation and generating more cracks in the rubberized concrete [35,36,37,38]. Although the inclusion of WRTCs aggregate slightly improved the residual CS attacked by acid solution, replacing sand or coarse aggregate with WRTCs aggregate by 5% significantly improved the CS of modified rubberized concrete attacked by sulphate solution compared with the control specimen prepared with OPC.

The concrete made from 20% of GBFS as the substitute to OPC showed the lowest CS loss after 365 days of immersion in both acid and sulphate. Such improvement can explain by GBFS strong chemical binding with the OPC matrix and the formation of the C-S-H gels, making the concrete network inaccessible to the sulphate ions, gypsum, and ettringite. This in turn, reduced the permeability and sulphate ions penetration within the concrete matrix [35,39]. It was also acknowledged that the amounts of Si, Al, and Mg might increase due to the addition of GBFS in the concrete, lowering the rate of gypsum development and improving the CS and the durability of the concrete [40,41].

Figure 2b also illustrates the weight loss percentage of all specimens after 365-days of MgSO_4_ and H_2_SO_4_ exposure. The results indicate that immersion in acid solution leading to a major weight loss compared to sulphate immersion. Modified rubberized concrete was exposed to MgSO4 solution experienced an average 8% weight loss, whereas this value increased by an average of 75% for H_2_SO_4_ immersion. Such reduction was ascribed to the diffusion of the acid inside the concrete matrix and subsequent destruction of the cement-gel binder. The chemical reaction between the generated soluble and soft Ca_2_SO_4_.2H_2_O with the calcium hydroxide was responsible for the formation of the ettringite, resulting in significant weight loss. Similar to CS reduction, there was a direct relationship between weight loss and the percentage of WRTCs aggregate. For example, increasing WRTCs aggregate from 5 to 30% led to an increase of around 3% in the weight deduction in all modified rubberized specimens exposed to MgSO_4_ and H_2_SO_4_ solutions. Besides, replacement of the OPC by 20% of GBFS led to an enhancement in the durability of the concretes with reduced deterioration. The amount of Ca in the concrete matrix was decreased due to the existence of GBFS, limiting the gypsum formation and causing lesser spalling and degradation [42].

**Figure 3:**
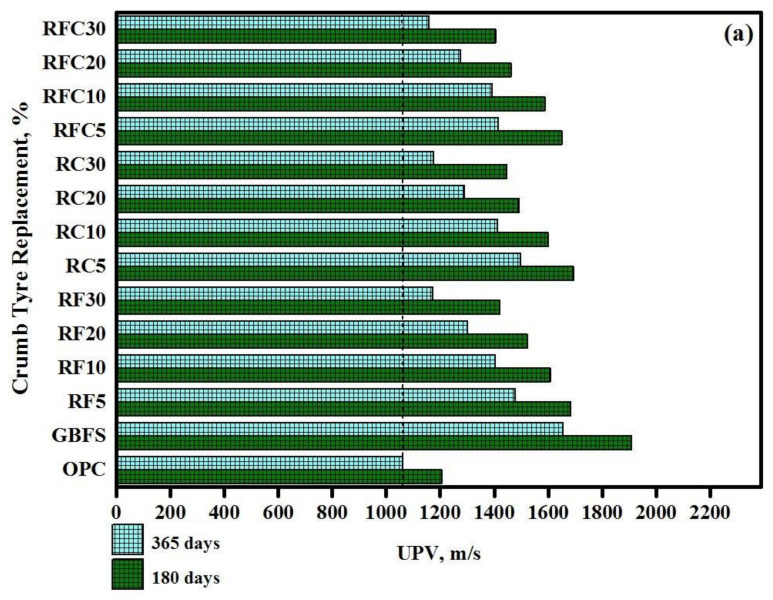

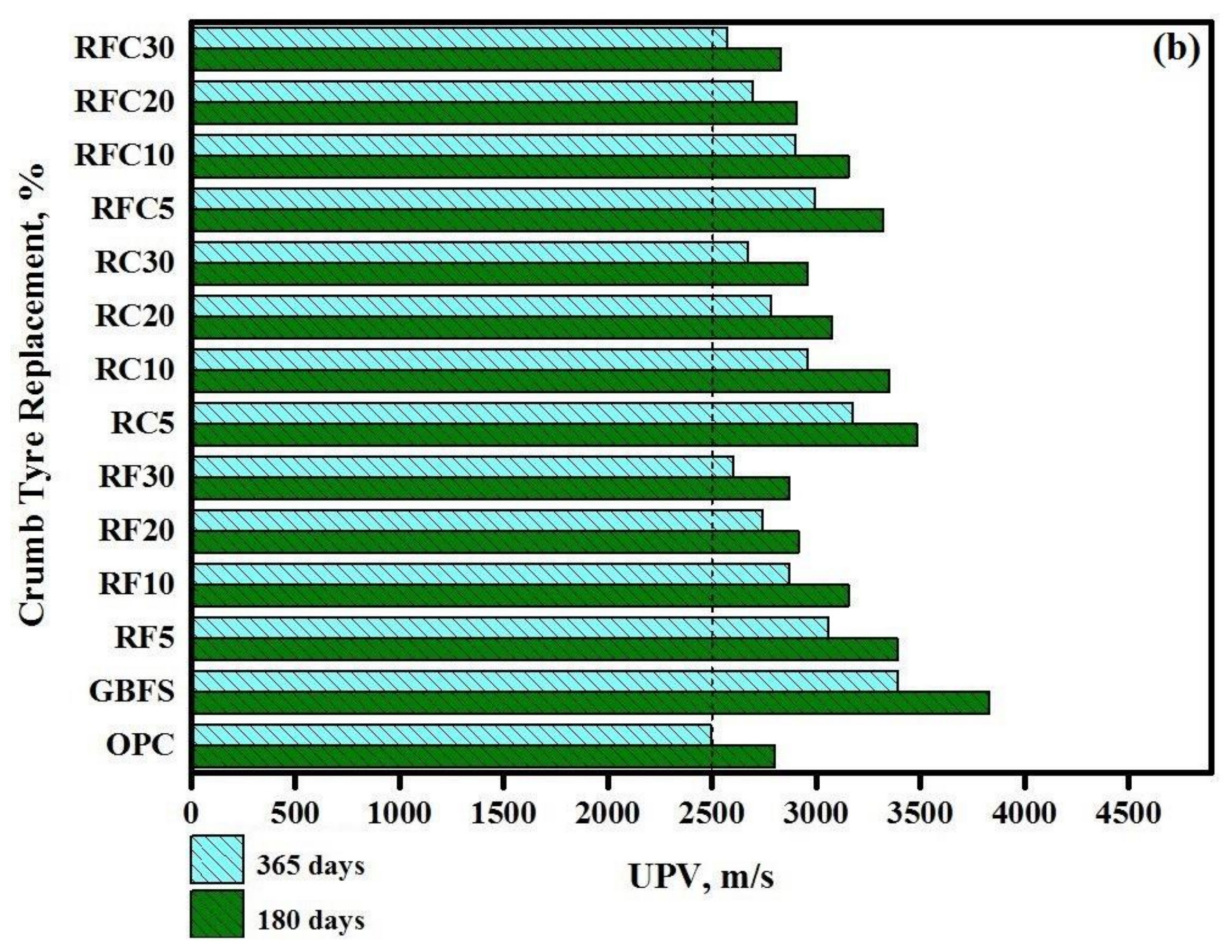
Ultrasonic pulse velocity (UPV) reading records of all studied specimens after 180 and 365-days of immersion in and (**a**) H_2_SO_4_ and (**b**) MgSO_4_ solutions.

Figure 3 displays the UPV results for all studied specimens after 180 and 365-days of immersion in MgSO_4_ and H_2_SO_4_ solutions. The results clearly showed that the pulse velocity in modified rubberized concrete expose to H_2_SO_4_ solutions was significantly lower compared to the velocity of specimens immersed in MgSO_4_ solution. Generally, a high value of UPV indicates a vice versa and denser microstructure.

The average pulse velocity at the age of 180-days in modified rubberized concrete expose to MgSO_4_ solution was 2.64, whereas this value declined to 1.35 after immersion in H_2_SO_4_ solutions. Meanwhile, the UPV was declined more than 15% and 45% after 365-days immersion in MgSO_4_ and H_2_SO_4_ solutions, respectively, compared to the velocity recorded at the age of 180-days. Similar to CS and weight loss, the pulse velocity also declined by increasing WRTCs aggregate in both sulphate and acid attack conditions. Such a decline in pulse velocity indications the likelihood of a higher number of cracks generation due to the rubber’s weak resistance against the expansion from the gypsum formation. This in turn, provided inadequate bond strength at the zones enclosing the modified rubberized pastes, thereby causing lowering the acid and sulphate attack resistance of the modified concretes containing higher amounts of WRTCs. It is claimed that the sulphate attacks can be led to the generation of expanding ettringite [3CaO-Al_2_O_3_-3CaSO_4_-32H_2_O] and gypsum [CaSO_4_-2H_2_O], causing the expansion or softening of the concretes.

The results also acknowledged that the design mix containing OPC provided the lowest pulse velocity in both acid and sulphate attack conditions, while by replacing 20% OPC with GBFS, the velocity significantly improved.

Previous literature indicated that the relationship between CS and pulse velocity as a measure of material deterioration, internal cracks, and/or pre-existing defects in the concrete before and after immersion in sulfuric acid and sulphate solutions could be estimated using the following exponential function [43,44,45].

(2) $$CS=Ae^{\left(BV\right)}$$

where *V* is the UPV, and the coefficients *A* and *B* are empirical constants.

**Figure 4:**
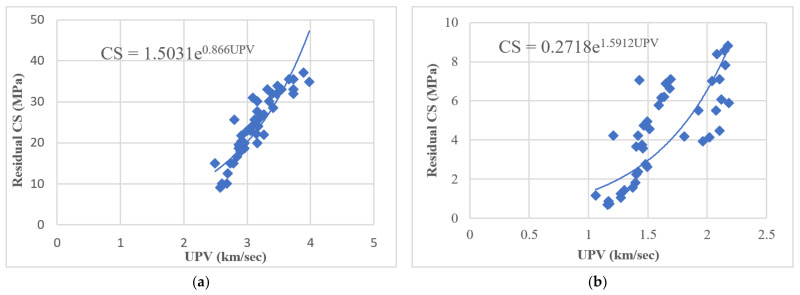
Relationship between UPV and residual CS for 14 studied specimens after immersion in (**a**) MgSO_4_ and (**b**) H_2_SO_4_.

Using nonlinear regression analysis, an exponential function for estimating the relationship between residual CS and pulse velocity of modified rubberized concrete was developed in this research. Figure 4 shows the relationship between UPV and CS’s mean values for all 14 studied specimens after 60, 180, and 365-days of immersion in MgSO_4_ and H_2_SO_4_ solutions.

**Figure 5:**
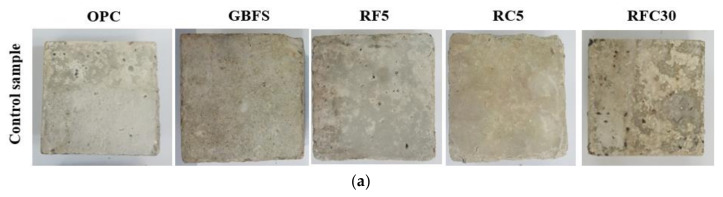

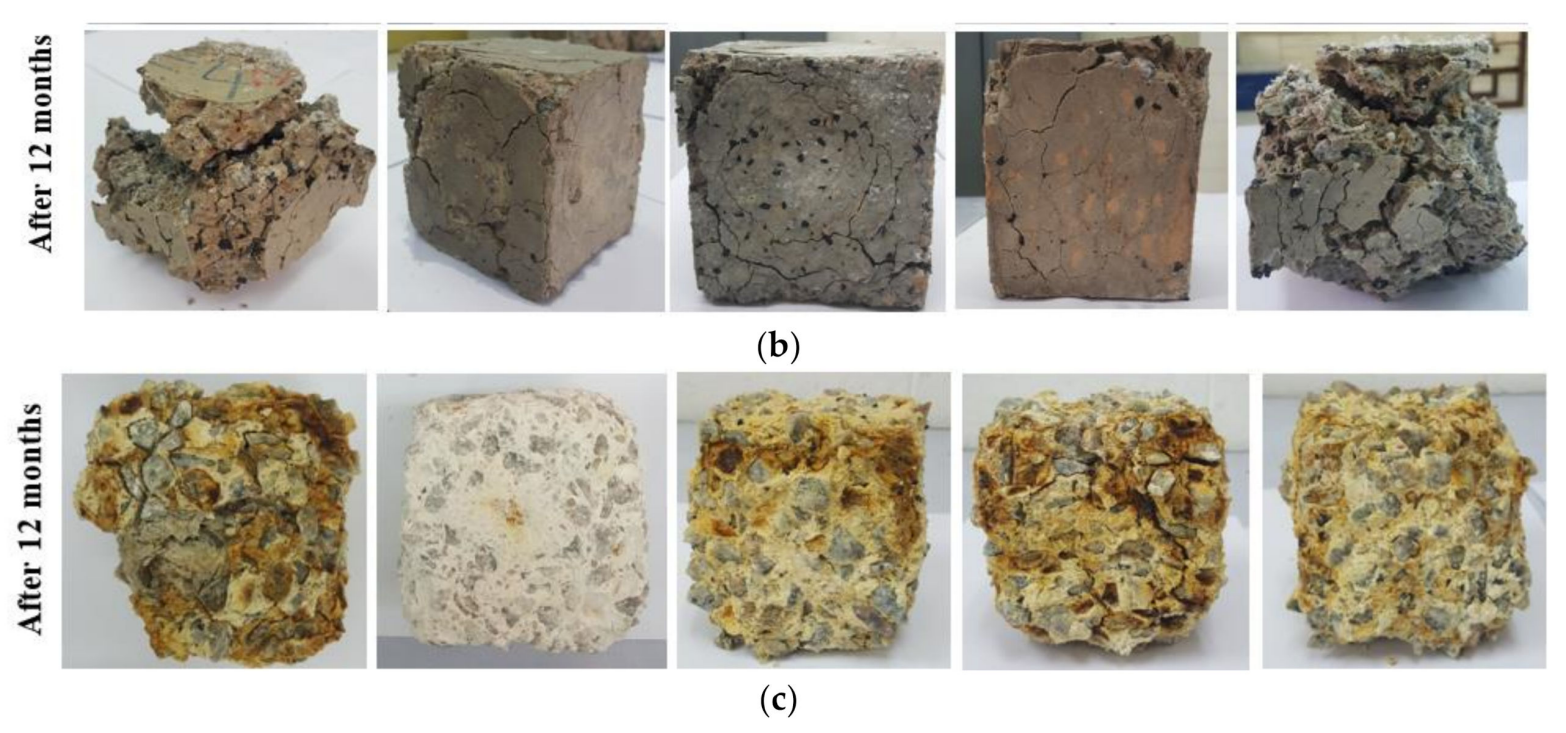
Surface texture of the (**a**) modified rubberized concretes, and after 365-days immersed in the (**b**) MgSO_4_ solution (sulphate), and (**c**) H_2_SO_4_ solution (acid).

Figure 5 presents the surface texture of the normal concrete, GBFS modified concrete and WRTCs concrete exposed to sulphate and acid environments for 365-days. The colors of the concrete provided important information regarding the effects of sulphate and acid attacks. After 365-days, a high deterioration was observed on the OPC, GBFS and WRTCs concretes’ surface. However, the deterioration rate on GBFS and 5% WRTCs was lower than that observed with OPC and 30% WRTCs specimens. The surface of the OPC and concrete with 30% of WRTCs showed large cracks in which increasing the WRTCs content, as fine or/and coarse aggregates, resulted in increase the crack formation. This was due to the loss of the bond between paste and WRTCs under high expansion from generated the gypsum and ettringite during the exposition period. According to Ganjian et al. [46] the low durability performance and strength loss of the rubberized concretes exposed to the sulphate solution may arise from various factors. First, the aggregates are enclosed in the cement pastes that contained the rubber aggregates. The cement pastes are softer than the concrete free from rubber particles where the cracks can occur and propagate faster around the rubber aggregates. In addition, once the gypsum and ettringite are formed, larger cracks in the concrete have high levels of WRTCs. Second, only few bonds are formed between the rubber particles and cement paste than the number of bonds between the cement paste and natural aggregate, yielding low durability of the rubberized concretes.

**Figure 6:**
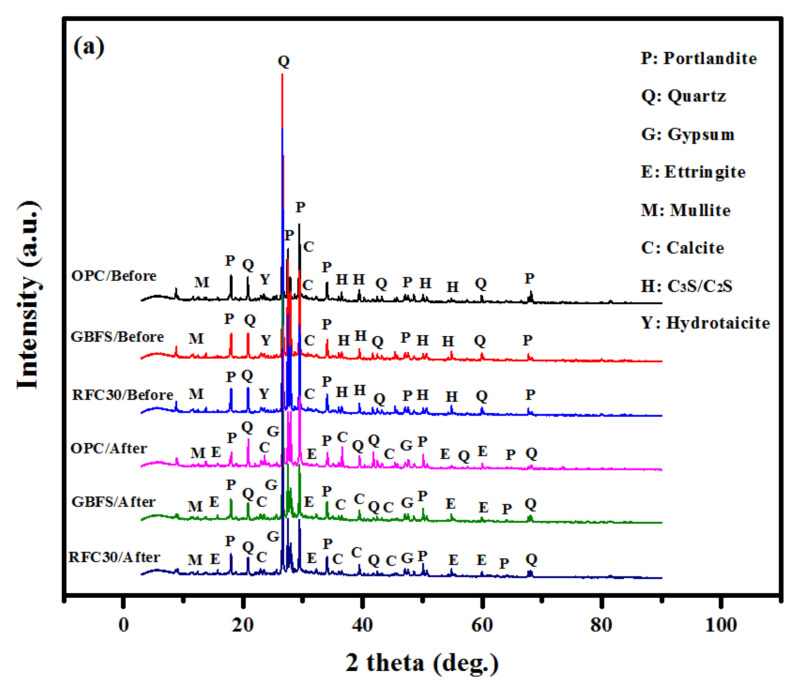

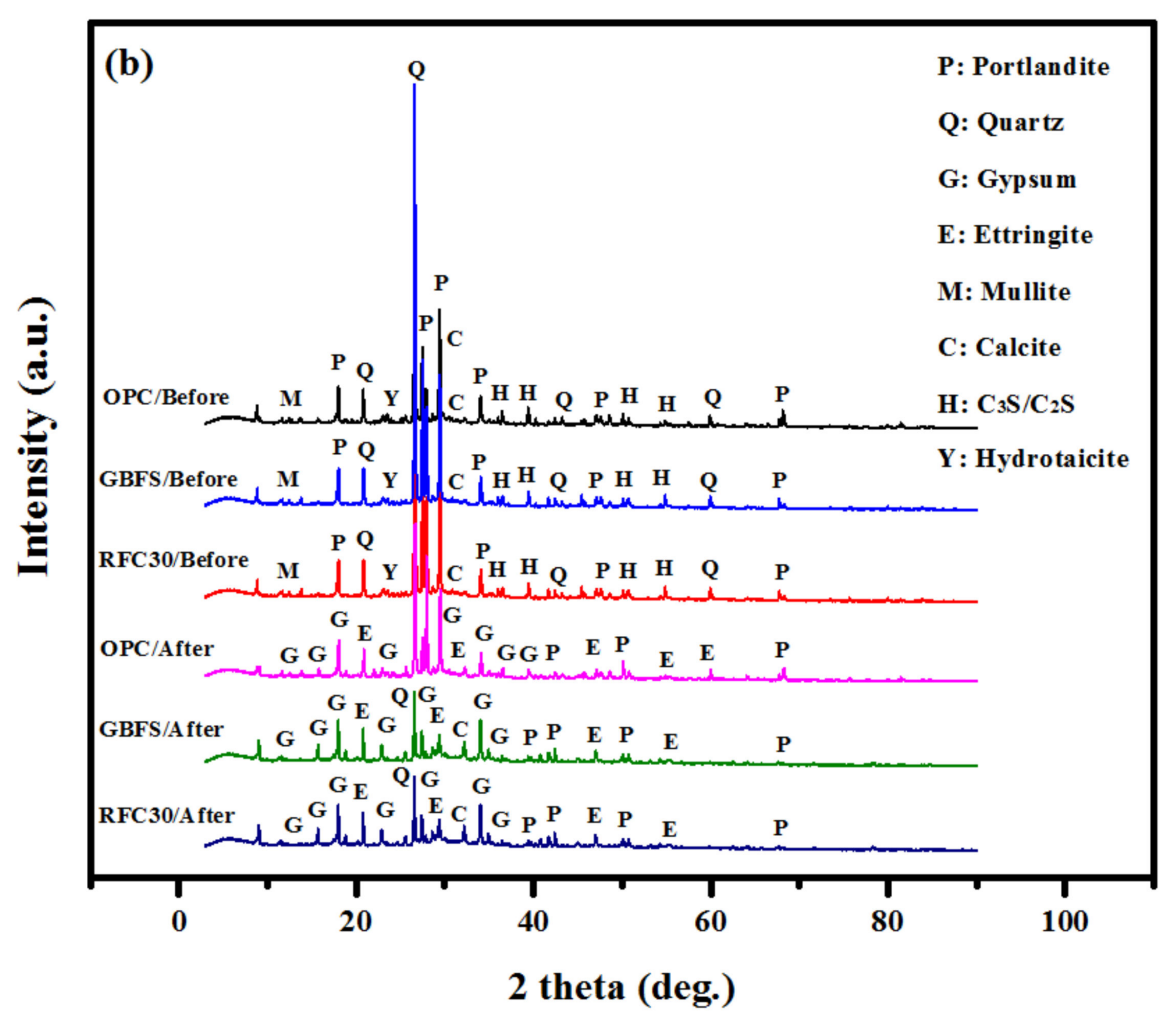
XRD patterns of the control (OPC), GBFS modified, and modified rubberized concrete (RFC30) (**a**) before and (**b**) after immersion in MgSO_4_ and H_2_SO_4_ solutions for 365 days.

Figure 6 illustrates the XRD patterns of the control (OPC), GBFS modified, and modified rubberized concrete (RFC30) specimens before and after immersion in 10% MgSO_4_ and 10% H_2_SO_4_ solutions for 365-days. Figure 6a indicated that the intensity of the peaks corresponding to the ettringite and gypsum in GBFS modified concrete was increased, indicating a better performance of the GBFS and RFC30 mixtures in the sulphate environment. Furthermore, the XRD data revealed a steady increase of gypsum in the OPC based concrete, indicating an expansive effect upon the formation. However, the XRD data showed the minimal change of ettringite or gypsum in the concrete containing GBFS immersed in MgSO_4_ solution after 365 days (GBFS and RFC30 mixtures). This observation was attributed to the lower amount of Portlandite in the GBFS mixtures that was consumed by the hydration products to form extra C-A-S-H gels, reducing the formation of the gypsum and ettringite [35].

Figure 6b indicates that the gypsum’s peak intensity appeared at 29.7° after immersion in the H_2_SO_4_ solution for 365-days. Additionally, the peak intensities of the gypsum at 11.8° and 18.2° were increased and moved nearer to the quartz peak at 27.6° at 27.9°. Bellmann and Stark [47] reported the difficulty in differentiating the quartz and gypsum peaks at 27.6 and 27.9°. A close view of these double peaks showed the presence of quartz and gypsum minerals in the modified GBFS concretes (GBFS and RFC30 mixtures). Once the GBFS content was raised to 20%, as a replacement for the OPC, it limited the gypsum formulation, which was indicated by the peak at 29.7°. Consequently, the durability performance and the resistance to the acid attacks of the concrete was improved. It was affirmed that the inclusion of the GBFS at high amounts in the concrete might be beneficial for the formation of extra C-A-S-H gels, responsible for resisting the sulphuric acid attack.

The calcium hydroxide within the OPC strongly reacted with the penetrated SO_4_^2−^ and produced gypsum (indicated by Equation (3)). The generated gypsum reacted with the acuminate calcium hydrate and formed ettringite (shown by Equation (4)), which led to the eventual failure through the crack formation.

SO_4_^2−^ + Ca(OH)_2_ → CaSO_4_ + 2OH^−1^ (3)

C_3_A + CaSO_4_ → Ca_6_Al_2_(SO_4_)_3_(OH)_12_·26H_2_O (4)

The degree of the concretes’ damage because of the H_2_SO_4_ solution exposure was correlated to the acid accumulation in the concrete matrix and exposure time span. The results revealed that the modified GBFS specimen had higher resistance against acid attack than the normal OPC concrete. The formation of CaSO_4_.2H_2_O (13.8%) and Ca_6_Al_2_(SO_4_)_3_(OH)_12_·26H_2_O (29.7%) in the GBFS modified specimen was lower than the OPC concrete (19.7% of gypsum and 13.9% of ettringite) as shown in Table 6. The occurrences of excess quantities of the gypsum and ettringite in the OPC based concrete were mainly responsible for the higher expansion of the matrix network and formation of more cracks. Consequently, the deterioration of the OPC based concrete in the acidic environment was more than the GBFS modified concrete.

**Figure 7:**
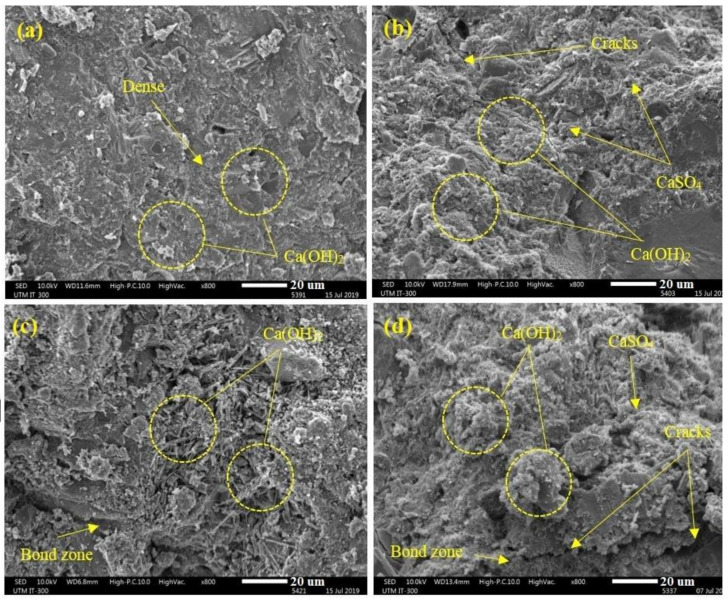
SEM micrographs of the (**a**) GBFS before (**b**) GBFS after (**c**) RF5 before and (**d**) RF5 after exposed to 10% of MgSO_4_ solution for 365 days.

Figure 7 depicts the SEM images of the GBFS and rubberized modified concrete (RF5) before and after 365 days MgSO_4_ solution exposure. The results indicate that the bond regions between the cement paste and natural aggregates were affected as a result of WRTCs inclusion. Additionally, larger cracks were observed in the RF5 specimen compared to the modified GBFS specimen.

**Figure 8:**
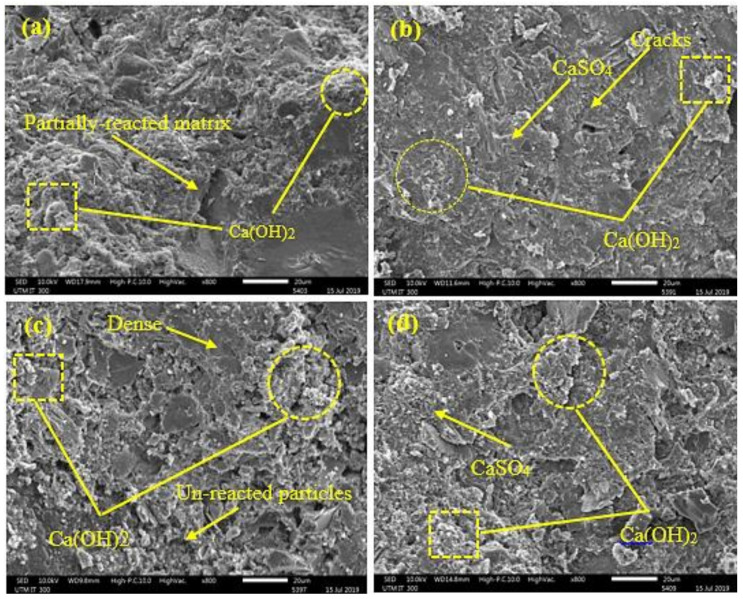
SEM micrographs of the (**a**) OPC based before (**c**) OPC based after, (**b**) GBFS modified before (**d**) GBFS modified after exposure to 10% of H_2_SO_4_ solution for 365 days.

Figure 8 displays the SEM micrographs of the OPC and GBFS modified concrete before and after 365-days exposure to the H_2_SO_4_ solution. The surface morphologies of the OPC-based concrete (Figure 8c) after immersion in H_2_SO_4_ solution exhibited a higher number of cracks than the GBFS modified concrete (Figure 8d), implying more gypsum and ettringite in the OPC specimen.

### 3.2. Resistance to Elevated Temperatures

**Figure 9:**
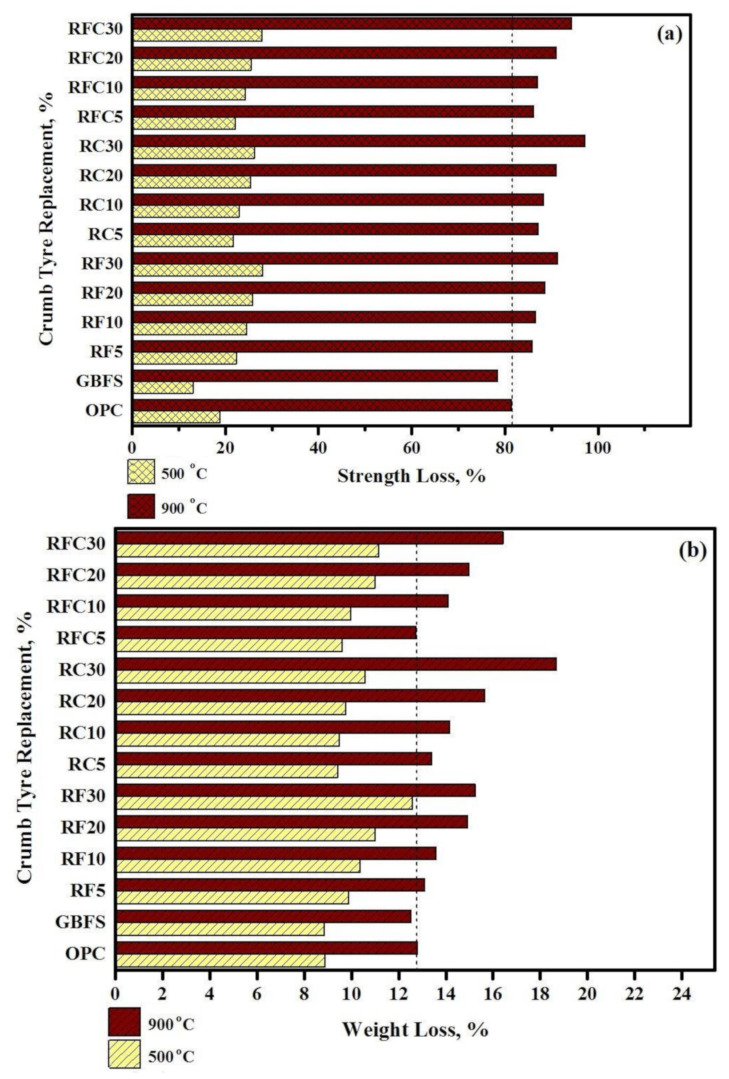
Effect of elevated temperatures on (**a**) CS and (**b**) weight loss of modified rubberized concrete.

Figure 9 shows the residual CS and weight loss of all tested specimens exposed to the 500 °C and 900 °C elevated temperatures. The results indicated that GBFS modified concrete provided the lowest CS and weight loss in both 500 °C and 900 °C elevated temperatures. This issue can be explained by the fact that the inclusion of GBFS in the concrete matrix led to the formulation of more calcite (CaCO_3_) and yielded high resistance to disbanding under elevated temperatures compared to the OPC matrix [48]. The results also acknowledged that all modified rubberized concrete specimens had higher residual CS and weight loss than control specimens. There was a direct relationship between increasing WRTCs and losing the residual CS and weight. For example, by increasing WRTCs content, as a fine or coarse aggregate, from 5 (RFC 5) to 30 (RFC 30), the residual CS and weight decreased by 9% and 29%, respectively, in the 900 °C elevated temperature. Such issue can explain by total melting of the WRTCs, sluggish evaporation of the water, dehydration of the C-A-S-H gels, and decay of Ca(OH)_2_ that occurred above 400 °C in the modified concretes [49].

Using multilinear regression, the following equations are proposed to estimate the residual CS and weight loss of modified rubberized concrete exposed to the 500 °C and 900 °C elevated temperatures.

(5) $$\%\;\mathrm{Loss}\;\mathrm{CS}\;\left(500\;T\right)=-1832+0.0474\mathrm{OPC}-0.0916\mathrm{Sand}+1.9\mathrm{Gravel}-0.0369\;\mathrm{WRTC}\;\mathrm{Fin}+2.0\;\mathrm{WRTC}\;\mathrm{Coarse}$$

(6) $$\%\;\mathrm{Loss}\;\mathrm{CS}\;\left(900\;T\right)=8080+0.0671\mathrm{OPC}-0.1225\mathrm{Sand}-8.0\mathrm{Gravel}-0.0601\;\mathrm{WRTC}\;\mathrm{Fine}-7.9\;\mathrm{WRTC}\;\mathrm{Coarse}$$

**Figure 10:**
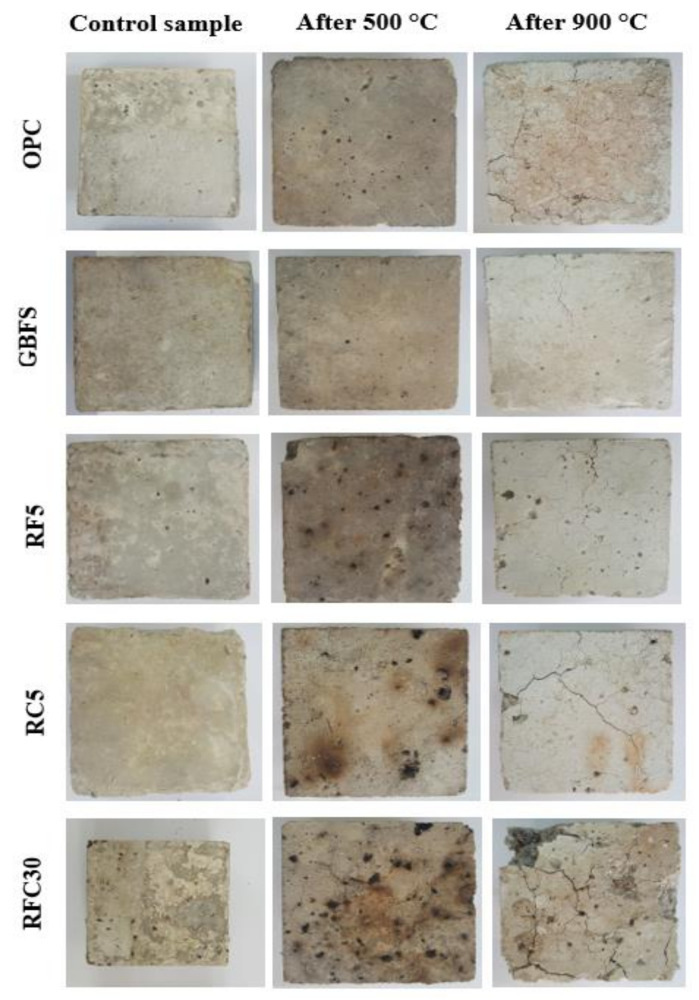
Surface texture of prepared concretes exposed to elevated temperatures.

Figure 10 shows the surface texture of prepared concretes exposed to 500 and 900 °C elevated temperatures. GBFS modified concrete showed less number of cracks and deterioration than pure OPC specimen. The inclusion of WRTCs in the concretes, as a fine/coarse aggregates replacement, produced lower resistance and a larger number of cracks at the elevated temperatures. Nevertheless, modified rubberized concrete can resist high temperature for at least 30 min before spalling due to its dense nature with high moisture content. Sancak et al. [50] stated that the primary reason for the spalling of concretes at high temperatures could be the internal pressure built up within the porous structure resulting from the free and bound water evaporation.

**Figure 11:**
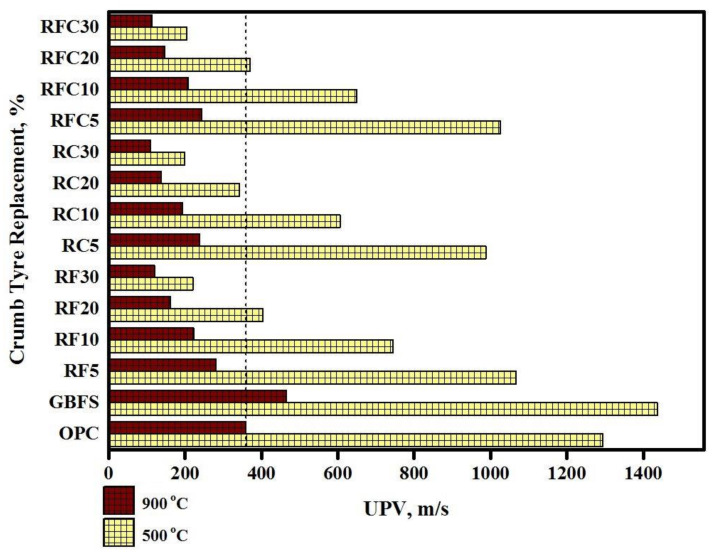
The UPV readings of the modified rubberized concrete exposed to the elevated temperatures.

Figure 11 shows the UPV readings of all tested specimens exposed to the 500 and 900 °C elevated temperatures. The results indicated that GBFS modified concrete provided the highest pulse velocity in both 500 °C and 900 °C elevated temperatures due to its dense structures. The results also acknowledged that all modified rubberized concrete specimens’ had lower pulse velocity than control specimen where the UPV was decreased by increasing WRTCs content. The lowest pulse velocity recorded in specimens with 30% WRTCs, as a fine or coarse aggregate, at the 900 °C elevated temperatures by around 0.11 km/s attributed to the decay of the C–S–H structures above 450 °C. Using multilinear regression, the following equations are proposed to estimate the UPV of modified rubberized concrete exposed to the 500 °C and 900 °C elevated temperatures.

(7) $$\mathrm{UPV}\;\left(500\;T\right)=804384-2.056\mathrm{OPC}+1.26\mathrm{Sand}-807\mathrm{Gravel}-4.44\;\mathrm{WRTC}\;\mathrm{Fine}-812\;\mathrm{WRTC}\;\mathrm{Coarse}$$

(8) $$\mathrm{UPV}\;\left(900\;T\right)=205028-1.793\mathrm{OPC}+2.32\mathrm{Sand}-207\mathrm{Gravel}+0.67\;\mathrm{WRTC}\;\mathrm{Fine}-208\;\mathrm{WRTC}\;\mathrm{Coarse}$$

**Figure 12:**
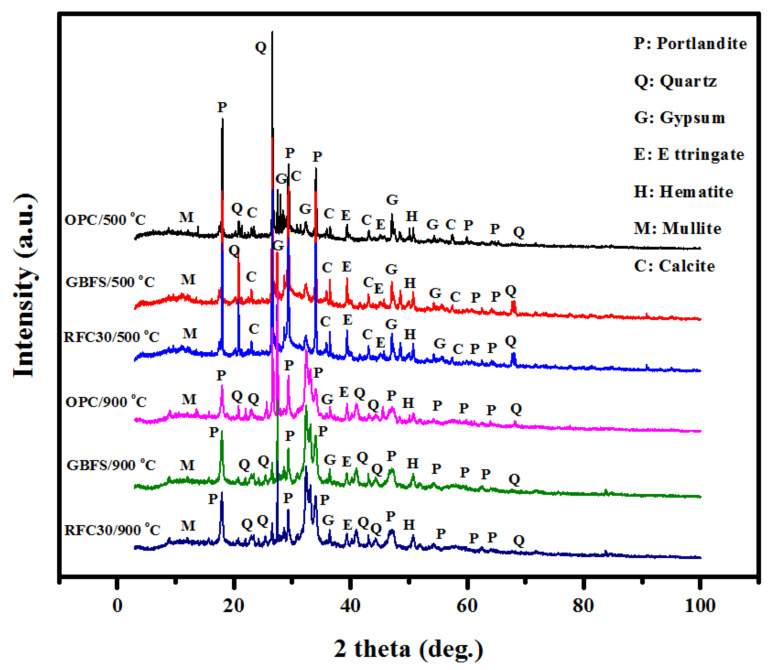
XRD pattern of the OPC based, GBFS, and rubberized modified concrete exposed to elevated temperatures.

Figure 12 illustrates the XRD patterns of OPC based, GBFS, and rubberized modified concrete after exposed to 500 and 800 °C elevated temperature. The XRD analysis demonstrated that the concretes composition was significantly affected by the fire or high temperatures. In all the above-mentioned specimens, the ettringite and gypsum were produced when subjected to 500 °C. Furthermore, the C-S-H and SiO_2_ peak intensities were significantly weaker for the heated treated mixes than those without elevated temperature exposure. Specimens exposed to heat measuring at 900 °C showed comparatively lower XRD peaks compared to specimens exposed to 500 °C heat, indicating that concrete decomposed at a higher rate with higher temperatures. However, the peaks at 18, 31, 37, 38, 41° and 48° for both OPC and GBFS specimens without heating were broader than those exposed to 500 °C. The breakdown which occurs within hydration products at 900 °C had a notable effect on the interface performance. Additionally, the dehydroxylation of calcium hydroxide is observable and results in the complete collapse of the concrete composition, as Ca(OH)_2_ was degraded and lost its place in the matrix, lowering the peak intensity of the C-S-H gel. The observed reductions may be due to the Portland dehydroxylation in the cement matrix, which was higher for OPC based concrete. This decay was responsible for the reduced performances and strength losses of the concretes at elevated temperatures. GBFS matrix possesses less calcite that leading to less dehydroxylation of the cement. Therefore, it can be concluded that the GBFS modified concrete provided higher fire resistance.

**Figure 13:**
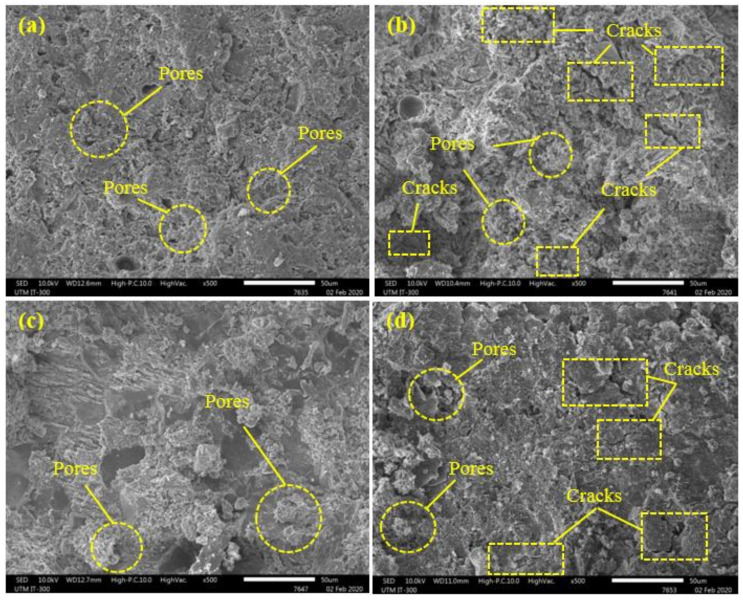
SEM image of control and GBFS modified concretes exposed to elevated temperatures (**a**) control specimen at 500 °C (**b**) control specimen at 900 °C (**c**) GBFS modified at 500 °C (**d**) GBFS modified at 900 °C.

Figure 13 displays the SEM images of the control specimen and GBFS modified concretes exposed to 500 °C and 900 °C elevated temperature. The SEM images revealed that GBFS modified concrete possessed little pores with continuous structure and devoid of micro-cracks at both500 °C and 900 °C elevated temperature compared to the control specimen with OPC. At elevated temperatures, the matrices of the GBFS showed higher resistance and performance compared to the OPC. However, at 900 °C, all the mixes’ microstructures were substantially damaged, leading to the decay of the C-S-H gels. Current observations are consistent with other reports [51,52].

**Figure 14:**
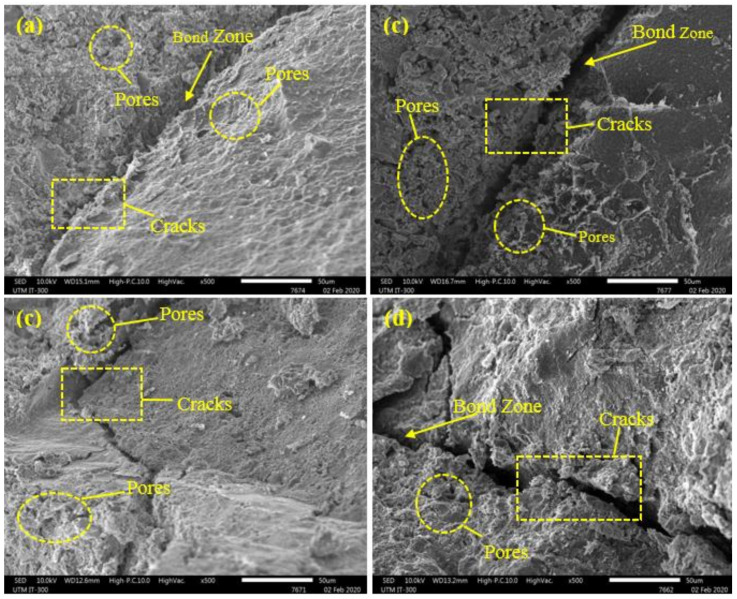
SEM image of modified rubberized concretes exposed to elevated temperatures (**a**) RFC 5 specimen at 500 °C (**b**) RFC 5 specimen at 900 °C (**c**) RFC 30 specimen at 500 °C (**d**) RFC 30 specimen at 900 °C.

Figure 14 displays the SEM image of the concretes containing WRTCs as the substitute for the fine and coarse aggregates exposed to 500 and 900 °C elevated temperature. For all concretes, the number of pores and cracks were increased with the increase in the temperatures. In addition, the results indicate that increasing WRTCs content, either fine or coarse aggregate, led to an increase in pores and cracks and showed more deterioration at 500 °C and 900 °C elevated temperature. However, design mixes containing WRTCs as the substitute for the coarse aggregates have a wider bond interface and more combustible under elevated temperatures, leading to a higher loss in bond strength and lower fire resistance performance as shown in Figure 14d [53].

## 4. Developing an Artificial Neural Network to Estimated CS Lose after Sulphate and Acid Immersion

Since the residual CS is the fundamental parameter in the harsh environment, an optimized artificial neural network (ANN) combined with the particle swarm optimization (PSO) was developed to estimate the residual CS of modified rubberized concrete after immersion in MgSO_4_ and H_2_SO_4_ solutions.

The multilayer feed-forward network provides a reliable feature for the ANN structure and, therefore, was used in this research. The multilayer feed-forward network comprises three individual layers: the input layer, where the data are defined to the model; the hidden layer/s, where the input data are processed; and finally, the output layer, where the results of the feed-forward ANN are produced. Each layer contains a group of nodes referred to as neurons that are connected to the proceeding layer. The neurons in hidden and output layers consist of three components; weights, biases, and an activation function that can be continuous, linear, or nonlinear. Standard activation functions include nonlinear sigmoid functions (logsig, tansig) and linear functions (poslin, purelin) [54]. Once the architecture of a feed-forward ANN (number of layers, number of neurons in each layer, activation function for each layer) is selected, the weight and bias levels should be adjusted using training algorithms. One of the most reliable ANN training algorithms is the backpropagation (BP) algorithm, which distributes the network error to arrive at the best fit or minimum error [55,56].

### 4.1. Particle Swarm Optimization

PSO is a population-based optimization technique inspired by the motion of bird flocks and schooling fish. This optimization technique has many similarities with evolutionary computation methods. PSO is developed initially by a population of random solutions, and subsequently search for the optimum solution through updating generations. Unlike genetic algorithm, PSO has no evolution operators including mutation and crossover and the possible solutions, particles, pass in the domain of the problem following the current optimal particles. This technique has efficiency of both memory requirements and speed.

In PSO, a candidate solution called a particle whereas a set of candidate solutions is referred to a swarm. A particle *i* can be defined by three vectors: *x_i_* (position), *v_i_* (velocity), and *x_i,Best_* (personal best position). The particle moves over the search domain defined by its own best known position, *x_i,Best_*, and the best known position of the whole swarm, *x_Best_*. The velocity of the particle can be calculated using following equations.

(9) $$v_{i,new}=c_{0}v_{i}+c_{1}r_{1}\left(x_{i,Best}-x_{i}\right)+c_{2}r_{2}\left(x_{Best}-x_{i}\right)$$

Subsequently the position updated using following equation:

(10) $$x_{i,new}=x_{i}+v_{i,new}$$

where *c*_0_ is the inertia weight, *r*_1_ and *r*_2_ are random numbers generated from *U* (0,1), and *c*_1_ and *c*_2_ are the cognitive and social acceleration weights, respectively. However, the local best solution *x_i_,_LBest_* was used in this research instead of the global best solution, *x_Best_*, and therefore the Equation (10) was updated as follow [57].

(11) $$v_{i,new}=c_{0}v_{i}+c_{1}r_{1}\left(x_{i,Best}-x_{i}\right)+c_{2}r_{2}\left(x_{i,LBest}-x_{i}\right)$$

For a given individual, the local best solution can determine by the best-known position inside that particle’s neighborhood. In this research the PSO algorithm was used an adaptive random topology in which each particle randomly informs *K* particles (K usually set to 3) and itself (this particle may be chosen several time). In such topology, the connections between particles may randomly change once there was no improvement in the global optimum [57,58,59].

### 4.2. Generation of Training and Testing Data Sets

To train and develop a reliable ANN, the percentage of OPC, GBFS, sand, gravel, fine, coarse, and the age of specimens were taken into account on the basis of input variables. The statistical analysis also confirmed that the two output parameters (residual CS after immersion in MgSO_4_ and H_2_SO_4_ solutions) followed normal distribution, positively improve the modelling accuracy of ANN.

The number of hidden layers and the total number of neurons in the hidden layers in an ANN depends on the problem’s nature. Generally, the trial-and-error method is used to obtain the ideal architecture, the architecture that best reflects the characteristics of the laboratory data. In this research, an innovative method for calculating the number of neurons in hidden layers was taken into account, as shown in the following equation.

(12) $$N_{H}\leq 2N_{I}+1$$

where *N_H_* is the number of neurons in the hidden layers and *N_I_* is the number of input variables.

Since the number of effective input variables is seven, the empirical equation shows that the number of neurons in hidden layers can be less than 15. Therefore, several networks with different topologies, with a maximum of two hidden layers and a maximum of 15 neurons, were trained and studied in this study. The hyperbolic tangent stimulation function and Levenberg–Marquardt training algorithm were used in all networks. The statistical indices used to evaluate the performance of different topologies were Root Mean Squared Error (RMSE), Average Absolute Error (AAE), Model Efficiency (EF), and Variance Account Factor (VAF) that are defined as follows.

(13) $$MSE=\frac{1}{n}\overset{n}{\underset{i=1}{\sum }}{\left(P_{i}-O_{i}\right)}^{2}$$

(14) $$ME=\frac{1}{n}\sum_{i=1}^{n}\left(P_{i}-O_{i}\right)$$

(15) $$MAE=\frac{1}{n}\overset{n}{\underset{i=1}{\sum }}\left|P_{i}-O_{i}\right|$$

(16) $$RMSE={\left[\frac{1}{n}\overset{n}{\underset{i=1}{\sum }}{\left(P_{i}-O_{i}\right)}^{2}\right]}^{\frac{1}{2}}$$

After examining different topologies, it was found that the network with 7-5-6-2 topology has the lowest value of error in RMSE, AAE, EF, VAF and the highest value of *R*^2^ to estimate the two output parameters as shown in Table 7. It is necessary to mention that the error criteria for training and testing the data are calculated in the main range of variables and not in the normal range.

**Figure 15:**
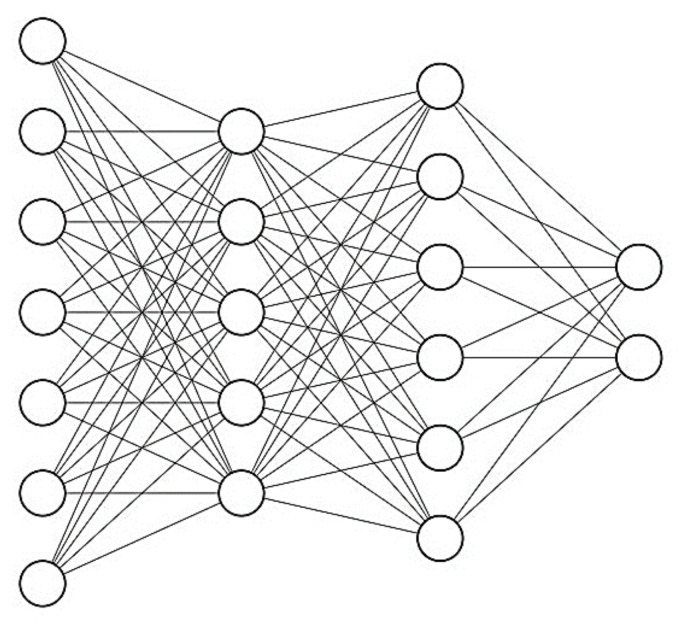
The topology of a feed-forward ANN with two hidden layers (7-5-6-2 structure).

Figure 15 shows the topology of a feed-forward network with two hidden layers, four input variables (neurons), and two output parameters.

The ANN used in this study was Newff Feed Forward. 80% of the experimental data (out of 54) was used for training, and the rest 20% was used for network testing. To optimize the ANN’s weights and biases, the PSO has been used to provide the least prediction error for trained structure. The properties of the PSO parameters are shown in Table 8, where lower and upper bound are the lower and upper limit of the network’s weight; swarm size refer to particle population size; max iteration refer to the number of algorithm’s iteration; C1 is a constant parameter that demonstrate the maximum distance between particle and optimized local position; and C2 is a constant parameter that demonstrate the maximum distance between particle and optimized global position.

### 4.3. Results

**Figure 16:**
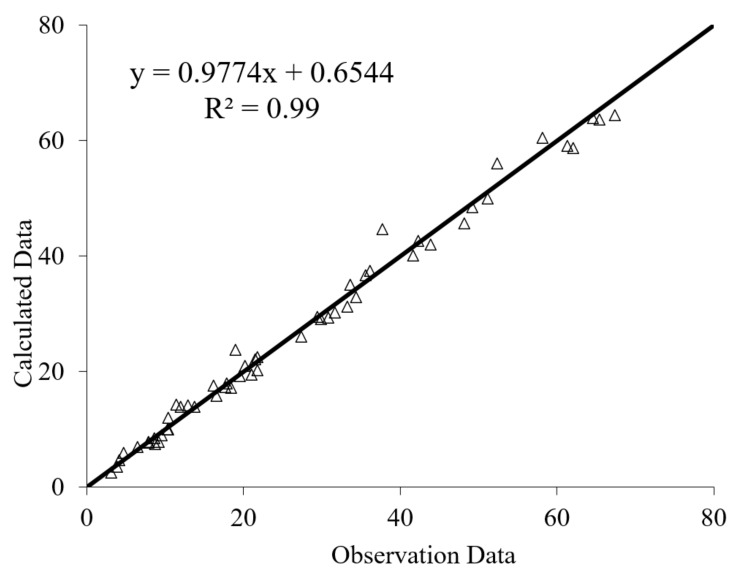
Predicted versus experimental values of residual CS after immersion in MgSO_4_ solution.

**Figure 17:**
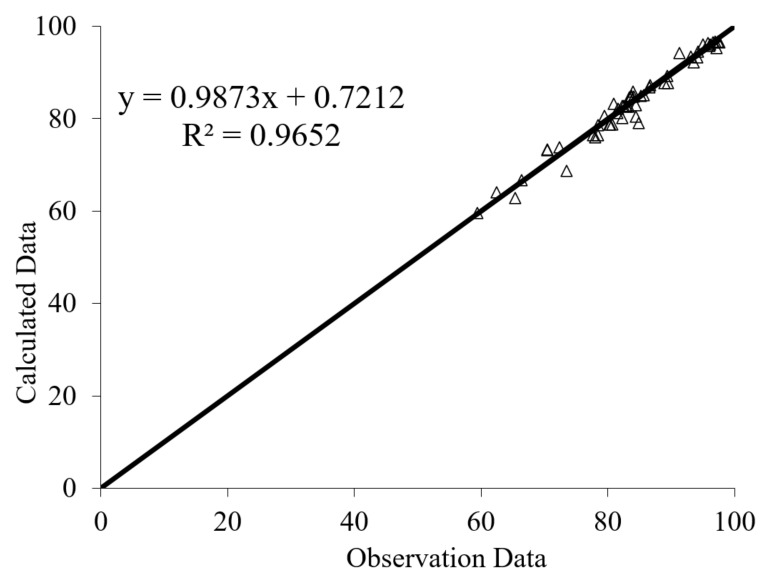
Predicted versus experimental values of residual CS after immersion in H_2_SO_4_ solution.

The results of PSO-ANN models are shown in Figure 16 and Figure 17 for residual CS after immersion in MgSO_4_ and H_2_SO_4_ solutions, respectively. The results indicate that the PSO-ANN estimated a reliable result for the ratio of observational to computational values, R^2^, for both input parameters, indicating the high potential and accuracy of the proposed model.

Table 9 provide the final weights and biases for both hidden layers estimated by the PSO-ANN model. Using the values of the weights and biases between the different ANN layers, the two output parameters (residual CS) can be determined and predicted.

## 5. Conclusions

This study investigated the performance of several modified rubberized concretes by exposing them to aggressive environments i.e., acid, and sulphate attacks, elevated temperatures. To examine the effect of WRTCs aggregate content and size on mechanical properties and structural morphology, four batches (A, B, C, and D) were developed as high-performance, sustainable concrete. By using the available experimental test database, an ANN model combined by particle swarm optimization algorithm was presented to estimate the residual CS of modified rubberized concrete after immersion in MgSO_4_ and H_2_SO_4_ solutions. The following provides the main findings of this research. The results indicated that modified rubberized concrete prepared by 5 to 20% WRTCs as a substitute to natural aggregate, provided lower CS and weight lose expose to sulphate and acid attacks compared to control specimen prepared by ordinary Portland cement (OPC).

- The results indicate that the CS in modified rubberized concrete experienced a major reduction after immersion in acid solution (by average 95%) compared to sulphate solution (by average 53%). Such a significant reduction can explain by SO_4_^−2^ reaction with the Ca(OH)_2_ that produces CaSO_4_·2H_2_O, leading to an expansion of the paste matrix and extra cracks generation in the concrete.
- The XRD patterns showed an increase in ettringite and gypsum in GBFS modified concrete after immersion in MgSO_4_ and H_2_SO_4_ solutions, indicating a high durability performance in such aggressive environment.
- GBFS modified concrete provides the lowest CS loss 15 and 80 MPa in 500 and 900 °C elevated temperatures, respectively. However, by increasing the WRTCs content in modified rubberized concrete from 5 to 30%, the residual CS and weight decreased by 9 and 29% that related to melting of the WRTCs, sluggish evaporation of the water, dehydration of the C-A-S-H gels, and decay of Ca (OH)_2_ that occurred above 400 °C.
- The ANN combined with particle swarm optimization algorithm provided satisfactorily results to estimate the residual CS of modified rubberized concrete after immersion in MgSO_4_ and H_2_SO_4_ solutions. In addition, the particle swarm optimization algorithm can also be used as a powerful tool in optimizing ANN weights. By using the optimized weight and bias of PSO-ANN, it is possible to design modified rubberized concrete with targeted mechanical properties and simultaneously manage the consumption of waste materials.

## Figures and Tables

**Table 1:** The chemical compositions of the OPC and GBFS obtained from the XRF analyses.

Materials	Elements (Mass %)						
	CaO	SiO_2_	Al_2_O_3_	Fe_2_O_3_	MgO	K_2_O	LOI
OPC	67.8	17.6	4.5	3.4	2.2	0.3	1.7
GBFS	51.8	30.8	10.9	0.6	4.6	0.4	0.2

**Table 2:** Physical properties of the rubber aggregates compared to the natural one.

Material		Maximum Size (mm)	Specific Gravity	Water Absorption (%)
Natural aggregates	Fine	4.7	2.7	2.2
	Coarse	10	2.8	1.2
Rubber aggregates	Fine	4	1.3	-
	coarse	8	1.4	-

**Table 3:** The chemical and physical features of the WRTCs aggregates.

Chemical Compositions Mass %	
Acetone extract	10
Ash content	24
Carbon black	14
Rubber Hydrocarbon (RHC)	52
**Physical properties**	
Size of fine rubber, mm	1–4
Size of coarse rubber, mm	5–8
Heat loss, kgf/cm^2^	<1
Metal content, %	<0.5
Fibre content, %	<1

**Table 4:** The mix design of the granulated blast furnace slag (GBFS) and WRTCs modified concrete.

Mixes		Binder		WRTCs Aggregate		Natural Aggregates	
		OPC (kg/m^3^)	GBFS (kg/m^3^)	Fine Rubber (kg/m^3^)	Coarse Rubber (kg/m^3^)	River Sand (kg/m^3^)	Crushed Stone (kg/m^3^)
Batch A	OPC	419	-	0	0	721	995
	GBFS	335.2	83.8				
Batch B	RF5	335.2	83.8	18.2	0	684.95	995
	RF10			36.5		648.90	
	RF20			72.9		576.80	
	RF30			109.4		504.70	
Batch C	RC5	335.2	83.8	0	25.5	721	945.25
	RC10				51.1		895.5
	RC20				102.1		796.17
	RC30				153.2		696.5
Batch D	RFC5	335.2	83.8	9.1	12.8	702.77	970.12
	RFC10			18.2	25.5	684.95	945.25
	RFC20			36.5	51.1	648.90	895.5
	RFC30			63.8	89.3	612.85	845.75

**Table 5:** Methods and tested specimens informations.

No.	Test	Age, Day	Number of Samples	Notes
1	Acid	28	84 (6 for each mixture)	Evaluated after 365 days
2	Sulphate	28	84 (6 for each mixture)	Evaluated after 365 days
3	Elevated temperatures	28	126 (9 for each mixture)	Evaluated at 500 and 900 °C

**Table 6:** The XRD results of the OPC based and GBFS modified concrete subjected to the acid attack.

Mix Design	Weight (%)				
	Gypsum	Ettringite	Portland	Quartz	Others
OPC based concrete	19.7	13.9	12.3	34.5	1.6
GBFS modified concrete	13.8	29.7	22.9	31.8	1.8

**Table 7:** Statistical indices of artificial neural network (ANN) with different topology.

Network Topology	Training				Testing			
	MSE	ME	MAE	RMSE	MSE	ME	MAE	RMSE
ANN-PSO (7-5-6-2)	2.689	0.002	1.193	1.640	5.471	−0.740	1.846	2.339
ANN-PSO (7-8-6-2)	0.000	0.000	0.000	0.000	96.745	−3.688	6.559	9.836
ANN-PSO (7-4-7-2)	0.043	0.000	0.101	0.207	189.692	3.052	8.139	13.773
ANN-PSO (7-7-5-2)	0.000	0.000	0.000	0.000	537.116	−6.380	12.987	23.176

**Table 8:** Properties of PSO parameters.

Parameter	Value	Parameter	Value
Lower Bound	−1	Swarm Size	200
Upper Bound	1	Maximum Iteration	100
Cognition Coefficient (C1)	2	Social Coefficient (C2)	2

**Table 9:** Final weights and bias values of the optimum ANN-PSO model 7-5-6-2.

IW							b1
−0.5274	1.0940	0.1130	0.2984	−0.5184	−0.1860	−0.1587	−0.3396
0.0714	0.1071	0.1291	0.2456	−0.5523	0.1663	−0.5016	−0.0201
0.3507	0.1851	0.1027	0.4281	0.1688	0.4901	0.5762	0.7749
−0.0951	0.0294	−0.1359	−0.1078	−0.6578	−0.2505	0.2984	−0.7203
−0.1100	0.6376	0.1747	0.6571	−0.1104	0.2225	−0.0218	0.0496
**LW1**					-	-	**b2**
−1.0485	0.0214	0.0112	−0.5400	0.5249			0.4195
0.4383	−0.0296	0.8801	−0.7924	−0.8425			0.0452
0.2861	−0.1557	−0.5375	−0.2095	0.5081			−0.1088
−0.0628	0.1352	−0.2958	0.3030	0.2954			0.6771
0.0133	0.1122	0.5471	−0.4729	−0.0676			0.2375
0.0730	1.0582	0.6763	0.6170	0.2833			0.3055
**LW2**							**b3**
0.7316	−0.0601	−0.4191	−1.0144	0.7436	0.2015		−0.0182
0.0103	0.9972	0.0474	−0.3476	0.3334	−0.1799		−0.3690

IW: Weights values for Input Layer; LW1: Weights values for First Hidden; LW2: Weights values for Second Hidden Layer; b1: Bias values for First Hidden Layer; b2: Bias values for Second Hidden Layer; b3: Bias values for Output Layer.

## Data Availability

Data sharing not applicable.
